# Hypercalcemia in a 67-Year-Old Female Following the Use of Calcium Sulfate Beads: A Case Report and Review of Literature

**DOI:** 10.7759/cureus.21671

**Published:** 2022-01-27

**Authors:** Hussam R Alkaissi, Samy I McFarlane

**Affiliations:** 1 Internal Medicine, Kings County Hospital Center, Brooklyn, USA; 2 Internal Medicine, Veterans Affairs Medical Center, Brooklyn, USA; 3 Internal Medicine, State University of New York Downstate Medical Center, Brooklyn, USA; 4 Medicine and Endocrinology, University of New York Downstate Medical Center, Brooklyn, USA

**Keywords:** calcium sulfate beads, osteoporosis, orthopedic procedures, femoral fracture, clinical endocrinology, iatrogenic hypercalcemia, acute hypercalcemia

## Abstract

While the milk-alkali syndrome is traditionally viewed as the sole cause of exogenous hypercalcemia, the wide use of calcium sulfate (CS) in orthopedic procedures introduced another important item to be considered in the differential diagnosis. Calcium sulfate beads are increasingly used as void fillers and prophylactic measures to prevent postoperative hardware infections. However, hypercalcemia secondary to rapid calcium absorption from calcium sulfate beads is generally an underrecognized adverse effect and likely underreported. Furthermore, with calcium sulfate beads, hypercalcemia can dramatically present with alteration in mental status. In this report, we present a case of a 67-year-old female who underwent two orthopedic procedures, where calcium sulfate beads were used in both. The patient, on both occasions, developed significant hypercalcemia, manifested as agitation and suicidal thoughts, with each episode resolving after proper hydration and lowering of serum calcium. Also, in this report, we examined the literature and highlighted the female predominance in the reported cases, often manifesting in postoperative day (POD) 4. Given the acuity and severity of hypercalcemia, it is paramount to anticipate hypercalcemia, monitor serum calcium postoperatively to allow timely interventions, and avoid potentially serious complications.

## Introduction

Calcium sulfate (CS) beads are increasingly used in orthopedic procedures as a void filler and a mode of delivering high concentrations of antibiotics locally to prevent postsurgical hardware infection. However, since they are readily absorbable, they can cause transient hypercalcemia [1,2]. The rates of hypercalcemia have been different in several reports, but few published cases demonstrated severe and symptomatic sequelae. Here, we report a case of a patient who developed hypercalcemia twice, after two procedures, in which both CS beads were used to prevent infection.

## Case presentation

A 67-year-old female with a right-sided total hip replacement presented with right hip pain following a mechanical fall. In the emergency department, the patient was found to have shortened right lower extremity with external rotation. X-ray showed transverse fracture across the femoral shaft proximally and oblique fracture distally around the intramedullary part of the artificial hip. The patient underwent revision of right hip arthroplasty with long stem femoral component, mid-shaft femoral plate, and cerclage wires, receiving prophylactic antibiotics on the day of surgery (cefazolin and gentamicin) and one unit of packed red blood cells during surgery. In addition, 50 mL of calcium sulfate (CS) beads with vancomycin were applied at the surgical sites to fill defects and prevent infection.

Past medical history included osteoporosis on alendronate 70 mg weekly started one year before presentation, essential hypertension on amlodipine 10 mg daily, and depression controlled with escitalopram. She also had HIV on antiretroviral treatment (elvitegravir/cobicistat/emtricitabine and tenofovir), with a CD4 count of 150 cells/mL, attributed to poor compliance. Social history was significant for cigarette smoking, with a family history of hypertension.

**Figure 1:**
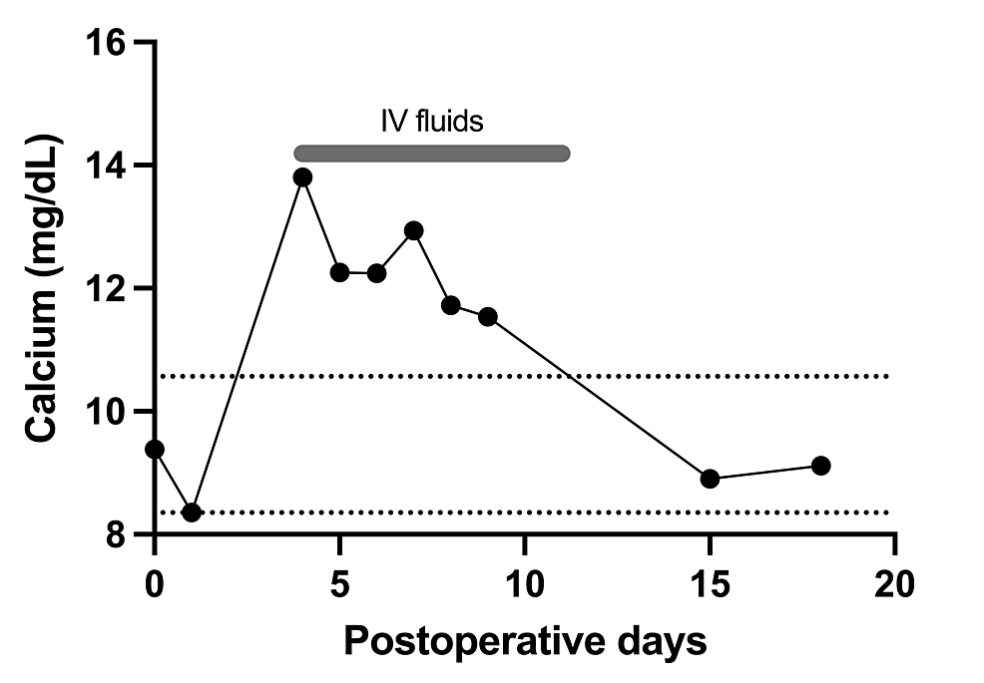
Calcium levels throughout the first hospitalization. Dashed line: normal range of calcium level, between 8.5 and 10.4 mg/dL; gray line: days receiving intravenous hydration for symptomatic hypercalcemia (postoperative day 4 to 11)

On postoperative day (POD) 4, the patient had a panic attack with suicidal ideation. She had no dyspnea, chest or abdominal pain, or polyuria. She was hemodynamically stable, and her physical examination was unremarkable apart from agitation. The metabolic panel on that day showed significantly elevated calcium, at a level of 12.6 (13.8 mg/dL corrected to albumin). Initially, no cause was found, as parathyroid hormone (PTH) levels were suppressed to 11 pg/mL (reference range: 15-65 pg/mL). Parathyroid hormone-related peptide (PTHrP) levels were at lower normal of 14 pg/mL (reference range: 14-27 pg/mL), and 25-OH cholecalciferol levels were also low at 17 ng/mL (reference range: 30-95 ng/mL). 1,25-Dihydroxycholecalciferol was also low at 8 pg/mL (reference: 18-78 pg/mL). Hydration with IV fluids started, and calcium was decreasing (Table 1 and Figure 1).

**Table 1:** Laboratory data over the first and second hospitalization. POD: postoperative day; PTH: parathyroid hormone; PTHrP: parathyroid hormone-related peptide

Variable	First admission	POD4	POD15	Second admission	POD4	Reference range
Sodium (mmol/L)	135	138	139	132	136	135–145
Potassium (mmol/L)	4.3	4.4	4.5	5.2	4	3.5–5.1
Chloride (mmol/L)	101	103	105	105	98	98–107
Carbon dioxide (mmol/L)	26	29	26	18	26	21–31
Urea nitrogen (mg/dL)	19	18	26	15	22	7–25
Creatinine (mg/dL)	1.3	1.1	1.7	1	1.1	0.6–1.2
Calcium (mg/dL)	8.9	12.6	8	8.1	10.6	8.2–10
Albumin (g/dL)	3.4	2.5	3	2	2	3.5–5.7
White cell count (per µL)	2,300	3,800	3,940	14,000	7,900	3,500–10,800
Hemoglobin (g/dL)	10.6	8.3	9	13	9.1	12–16
Platelet count (per µL)	158,000	139,000	176,000	164,000	186,000	130,000–400,000
PTH (pg/mL)		11				15–65
PTHrP (pg/mL)		14				14–27
25-OH vitamin D (ng/mL)		17				30–95
1,25-(OH)_2_ vitamin D (pg/mL)		8				18–78

**Figure 2:**
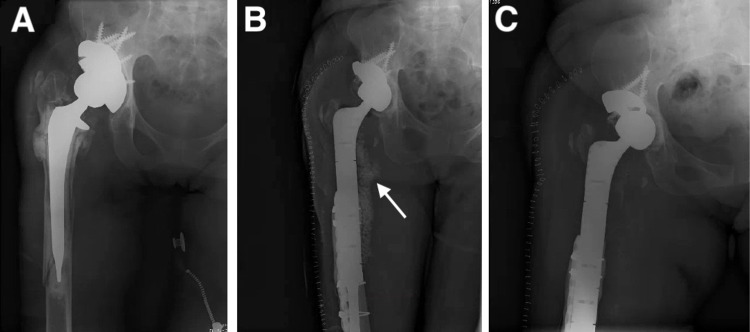
Serial X-rays of the right hip joint and femur. (A) On admission: X-ray of the right hip joint showing transverse fracture across the femoral shaft. (B) Postoperative day 3: replacement of intramedullary nail, with application of calcium sulfate beads seen as radio-opaque material on the medial aspect of the femur (arrow). (C) Postoperative day 9: complete absorption of calcium sulfate beads.

Serial X-rays showed gradual and rapid fading of the calcium sulfate bead opacity, with gradual improvement of hypercalcemia (Figure 2). In addition, the patient's mental status improved as the calcium levels normalized.

With intravenous hydration, renal function and calcium levels normalized, and the patient was discharged to rehabilitation. Three months later, the patient's rehabilitation course was complicated by a dislocation of the right hip joint, and she was readmitted for open reduction. CS beads with antibiotics were also used. With knowledge of prior adverse reaction, calcium levels were monitored daily, and interestingly, calcium levels rose from 9.7 mg/dL to 12.2 mg/dL over four days postoperatively. Earlier detection led to earlier proper hydration; thus, the patient was asymptomatic, and calcium level normalized on postoperative day 8, down to 10.1 mg/dL.

## Discussion

Prosthetic joint infection complicates 1%-2% of total hip and knee replacement surgeries. Biofilm-forming coagulase-negative *Staphylococcus* and *Staphylococcus aureus* cause the majority of these infections. Due to reduced vascularity of surfaces of prosthetic joints, treatment can be challenging once biofilm forms. As such, local antibiotics delivery methods have been implemented. Initially, polymethylmethacrylate (PMMA) beads were used to deliver high concentrations of antibiotics locally. Unfortunately, PMMA beads were nonabsorbable and thus had to be surgically removed once they served their purposes. They have been primarily replaced by readily absorbable calcium sulfate hemihydrate beads [1].

The use of calcium-containing material as a filler also dates back to the 1890s [3]. Later in 1959, Peltier experimented with calcium sulfate hemihydrate (plaster of Paris) as a filler. Initial experiments were run on dogs, and some dogs were transiently hypercalcemic after applying the calcium sulfate [4]. Later on, throughout the century, calcium sulfate gained popularity when it was shown that it could be mixed with antibiotics, leading to decrease prosthetic joint infections [2].

Some of the common complications seen after using CS beads are serous drainage from surgical sites, heterotopic calcification, and hypercalcemia [1]. The earliest two reports of hypercalcemia in human subjects date back to 2005. One by the FDA, in a 25-year-old male with systemic lupus erythematosus (SLE), developed transient hypercalcemia following CS bead use after a total hip replacement surgery [5]. In the same year, Smith reported a peculiar case of isolated increase of cerebrospinal fluid (CSF) calcium levels in a 69-year-old female who had an L4 and L5 decompression revision. CS beads were used, and on postoperative day 5, she was confused, developed myoclonus and seizures, then went into a coma. She was hypertensive at 200/110 mmHg with new-onset atrial fibrillation. Serum calcium was normal at 8 mg/dL, but CSF calcium was 19 mg/dL (expected would be 40%-60% of serum calcium). She made a full recovery 10 days later. It was postulated that calcium leaked into the CSF directly due to proximity [6].

In a study of 755 patients, Kallala et al. found that 41 (5%) patients developed transient hypercalcemia. The majority were asymptomatic, except for two female patients [1]. Since then, several other cases have been reported in the literature (Table 2) [7-21].

**Table 2:** Summary of published cases (individual cases, case series, and adverse event profile from larger studies). AKI: acute kidney injury; CSF: cerebrospinal fluid; NA: data not available; POD: postoperative day; AMS: altered mental status

Author	Year	Gender	Age (years)	Clinical	Calcium (mg/dL)	Bead volume (cc)	Duration (days)	Treatment
Smith [6]	2005	Female (1)	69	POD5: confusion, seizures, coma, and hypertension; high calcium in CSF due to direct leak	19 (CSF)	NA	3	Hydration, furosemide, calcitonin, and pamidronate
FDA [5]	2005	Male (1)	NA	NA	NA	NA	NA	NA
Kallala et al. [7]	2015	Male (1)		POD2: confusion and lethargy	14.1	40	10	Hydration and zoledronic acid
Carlson et al. [8]	2015	Female (1)	72	POD5: delirium	14.5	NA	NA	Hydration and calcitonin
Truong et al. [9]	2017	Male (1)	88	POD6: agitation and confusion	14.5	40	5	Hydration, calcitonin, and pamidronate
Kallala et al. [1]	2018	Females (2)	NA	NA	14.9 and 14.2	NA	NA	NA
Zozobrado et al. [10]	2019	Male (1)	58	POD2: AKI	13.6			Hydration, calcitonin, and pamidronate
Ma et al. [11]	2019	Male (1)	59	POD4: asymptomatic hypercalcemia; POD5: AKI secondary to high serum vancomycin	10.9	NA	NA	NA
Vora et al. [12]	2019	Female (1)	58	POD2: AMS, respiratory failure, and AKI	15.7	NA	10	Hydration, CRRT for eight days, and hemodialysis for two days
Magdaleno et al. [13]	2019	Female (1)	61	POD3: AMS and azotemia	17.4	10		Hydration and calcitonin
Challener et al. [14]	2019	Female (1)	90	POD7: confusion and AKI	13.4	NA	NA	Hemodialysis for three days
El-Bahri et al. [15]	2019	Female (1)	68	POD5: acute encephalopathy	13.5	30	7	Hydration, calcitonin, and pamidronate
Kuo et al. [16]	2019	Female (1)	69	POD9: AMS, AKI, and acute pancreatitis	16.1		5	Hydration, furosemide, and calcitonin
Jung et al. [17]	2020	Female (1)	58	POD2: confusion, hypertension, and AKI	21.1	50	2	NA
Gilbert [18]	2020	Female (1)	58	NA	>22		NA	Hydration, bumetanide, and pamidronate
Lane et al. [19]	2020	Five patients	NA	NA	10.7–16.1	10–60	14–21	Hydration, furosemide, calcitonin, denosumab, and zoledronic acid; one required hemodialysis
Garcia et al. [20]	2020	Female (1)	86	POD12: delirium	11.6		4	Hydration
Motevalli et al. [21]	2020	Female (1)	63	POD5: confusion and AKI	13.7	30		Hydration, calcitonin, and pamidronate

Upon literature review, we found that onset of symptoms varied between postoperative days 2 to 12, the majority within the first five days postoperatively. The average serum calcium was 15.1 mg/dL. IV hydration, furosemide, calcitonin, and bisphosphonate were the common treatment modalities. Symptoms resolved within two to 10 days after treatment of hypercalcemia. Interestingly, we found a significant gender difference, where most of the cases reported are females (14 (65%) females versus five (35%) males).

Hypercalcemia results from an imbalance between calcium released to the circulation and the urinary excretion rate. As a result, an increase in calcium entry to the circulation or a decrease in renal clearance of calcium can cause significant hypercalcemia. While primary hyperparathyroidism remains the most common cause of hypercalcemia (accounting for 90% of cases), other causes of hypercalcemia remain relatively common in clinical practice.

Here, we reported a case of transient hypercalcemia in another female patient, occurring twice after application of CS beads, once for hardware replacement and then after open reduction of a dislocated hip. On both occasions, peak calcium levels were recorded on postoperative day 4. The patient's total serum calcium was normal on the procedure day (8.9 mg/dL) with normal kidney function (eGFR of 100 mL/minute/1.73 m^2^). However, on postoperative day 4, the patient developed severe symptomatic hypercalcemia (total calcium: 12.6 mg/dL) with altered mental status in the setting of low PTH (11 pg/mL) and 25-OH vitamin D (17 ng/mL), pointing toward a non-PTH-driven cause of hypercalcemia. Of note, low albumin was attributed to poorly controlled HIV infection due to nonadherence with antiretroviral treatment, and calcium levels were corrected to albumin levels.

Furthermore, PTHrP and 1,25-(OH)2 cholecalciferol were low. Other potential causes of hypercalcemia include immobilization-induced hypercalcemia. Immobilization usually results in hypercalcemia in patients with spinal injury weeks after immobilization. In a study of 11 healthy subjects, immobilization for 12 weeks resulted in a minor increase in serum calcium from 9.3 to 9.5 mg/dL throughout the immobilization, but urinary calcium increased five weeks after immobilization [22]. Our patient developed hypercalcemia four days after the surgery, and she was not entirely immobile, as she could sit at the bedside and on a chair. Similarly, once the CS beads were used again for the second surgery, a similar rise in calcium was noticed, pointing toward the CS beads as a potential cause rather than immobilization. One weakness in our case is that we did not collect urine to measure calcium excretion, as it should correlate with the volume of CS beads applied (50 cc).

According to the manufacturer, the calcium sulfate-based beads are mixed with antibiotics, and the gradually dissolving calcium sulfate allows a steady release of antibiotics and subsequently increases the zone of coverage [23]. Possible hypotheses as to why hypercalcemia affects some patients, but not others, may be related to patient factors or CS bead-related factors. Specific batches might have a defective calcium release, leading to rapid absorption. Carlson et al. were surprised to see that the CS bead opacity was completely vanished five days after its application, correlating with the hypercalcemia in their patient, indicating rapid absorption [8]. Other possible factors may include which kind of antibiotics are mixed with the calcium sulfate hemihydrate. The three commonly used antibiotics are vancomycin, gentamicin, and tobramycin. There had been cases of rapid release of vancomycin from CS beads, with related acute kidney injury [14].

**Figure 3:**
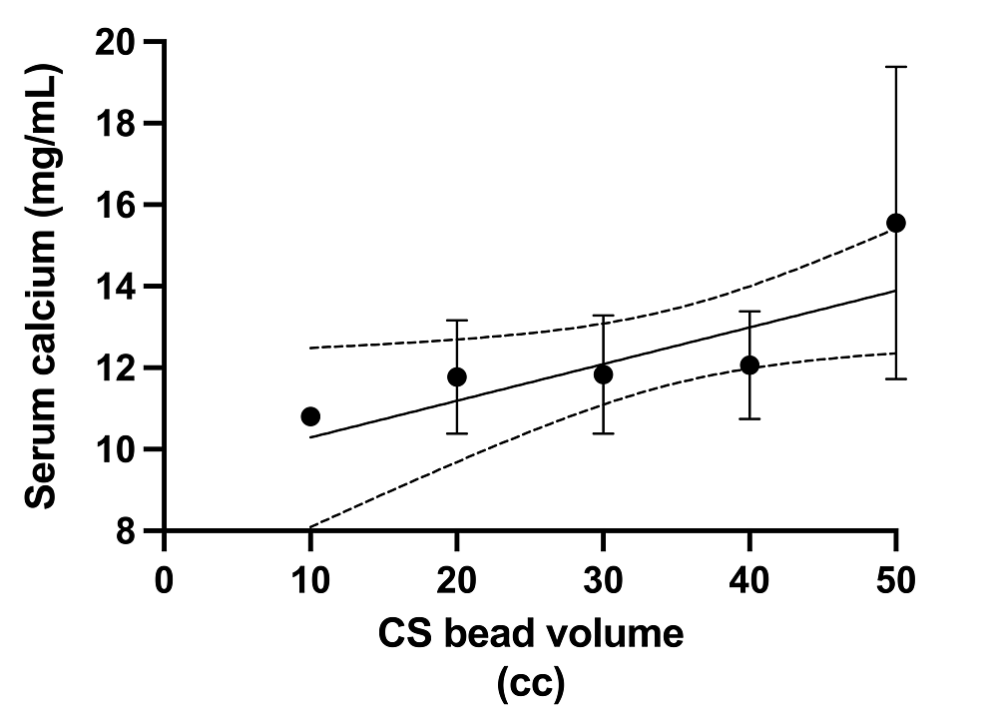
Correlation between the volume of applied CS beads and the degree of hypercalcemia. Data were presented as mean (dots) and SEM (whiskers) from several case reports [7,9,13,15,17,21]. CS: calcium sulfate; SEM: standard error of the mean; line: linear correlation, with confidence intervals presented as dotted lines R2 = 0.2, p = 0.03

Bead volume seems to correlate with the degree of hypercalcemia. We further looked at the nine patients reported by Kallala et al. and case reports that include bead volume [7,9,13,15,17,21]. Simple linear regression showed a significant correlation between bead volume and degree of hypercalcemia (R2 = 0.2, p = 0.03) (Figure 3). Therefore, to avoid hypercalcemia, the current recommendation is to use less than 40 cc of CS beads [1].

Other possible causes might be patient-related. Applying the CS beads near rich vascular beds may enhance calcium release into the circulation. As mentioned earlier, most of the reported symptomatic cases are females (about 65%). In fact, in a recent study done in a predominantly male population (84% were males), serum calcium mildly decreased postoperatively and after applying CS beads. Although it was not statistically significant, serum calcium decreased from 9.2 to 8.8 mg/dL [24]. There might be a gender difference in handling calcium. The term "calcigender" was recently used to describe such differences in calcium homeostasis. Females of many species, including humans, have been shown to extrude calcium, partly evolutionary for fetal skeletal development. Recent insight into calcium channels showed that estradiol increases calcium absorption through transient receptor potential vanilloid 5 (TRPV5) and TRPV6, major channels in the kidneys, and other tissues [25]. Furthermore, 17-β estradiol leads to increase mRNA expression of TRPV6 [26].

## Conclusions

In this case, we would like to draw attention to the possibility of symptomatic, severe hypercalcemia following the application of calcium sulfate beads used in orthopedic procedures as fillers and mode of antibiotic delivery. Therefore, it is crucial to monitor calcium levels postoperatively for early detection and treatment of hypercalcemia. In addition, it is important to follow recommendations of applying less than 40 cc of calcium sulfate beads if possible to avoid symptomatic hypercalcemia.
